# Novel Fault Diagnosis of a Conveyor Belt Mis-Tracking via Motor Current Signature Analysis

**DOI:** 10.3390/s23073652

**Published:** 2023-03-31

**Authors:** Mohamed Habib Farhat, Len Gelman, Abdulmumeen Onimisi Abdullahi, Andrew Ball, Gerard Conaghan, Winston Kluis

**Affiliations:** 1 School of Computing and Engineering, The University of Huddersfield, Queensgate, Huddersfield HD1 3DH, UK; 2 Daifuku Airport Technologies, Sutton Road, Hull HU7 0DR, UK; 3 Babcock International Group, Schiphol Boulevard 363, 1118 BJ Schiphol, The Netherlands

**Keywords:** diagnosis, conveyor belt, data analysis

## Abstract

For the first time ever worldwide, this paper proposes, investigates, and validates, by multiple experiments, a new online automatic diagnostic technology for the belt mis-tracking of belt conveyor systems based on motor current signature analysis (MCSA). Three diagnostic technologies were investigated, experimentally evaluated, and compared for conveyor belt mis-tracking diagnosis. The proposed technologies are based on three higher-order spectral diagnostic features: bicoherence, tricoherence, and the cross-correlation of spectral moduli of order 3 (CCSM3). The investigation of the proposed technologies via comprehensive experiments has shown that technology based on the CCSM3 is highly effective for diagnosing a conveyor belt mis-tracking via MCSA.

## 1. Introduction

Given their sustainability, efficiency, and high performance, belt conveyor systems present one of the most popular types of conveyors used in industries. They are widely employed in various applications such as airports, mining, manufacturing, transportation, the chemical industry, and the building materials industry. They are used to transport bulk materials, packages, and parcels, as they save time and labor and can be used to transport a wide variety of objects of different shapes and sizes.

Typically, the conveyor belt systems are composed of a belt tensioned by means of two or more pulleys. The belt is looped around the pulleys, allowing it to rotate continuously. One pulley, called a drive pulley, powers or pulls the belt, carrying the items from one place to another.

Belt mis-tracking, belt slippage, belt tear, belt deviation, and motor, gearbox, and idler faults are the most common defects encountered by belt conveyors in industrial applications. The idler and gearbox diagnosis of belt conveyor systems were achieved in Refs. [1,2] and Refs. [3,4,5,6] via vibration analysis. The wavelet transform was used to decompose the signals for diagnosis, such as in Refs. [7,8], and afterward, the energy of every frequency band is computed. The energies are used as features for the classification of data to diagnose idler faults. Magnetic field variation around defects was used in Ref. [9] to detect cuts, broken wires, and corrosion of cords in the core of steel-cord belts. A similar technique was utilized for monitoring the condition of splices between the belt pieces of conveyors, Ref. [10]. The belt and motor fault diagnosis of a belt conveyor system was achieved in Ref. [11] via a dataset, which was extracted using a coal mine monitoring information system. Ref. [12] proposed energy-based prognostics for the gradual loss of conveyor belt tension in discrete manufacturing systems. Ref. [13] investigated the effect of operational factors on conveyor belt mechanical properties. In Ref. [14], the authors proposed a new method for the predictive replacements of belt sections in a conveyor belt loop with their refurbishment, based on their conditions, recognized by specially designed diagnostic devices.

Mis-tracking is the tendency of a belt to pull to one side and to run off its intended track. In an industrial application, belt mis-tracking is a significant problem when running a belt conveyor system. A belt, which keeps tracking off a center, causes the edge of a belt to become damaged. This can generate uneven belt wear, belt damage/failure, and an essential disruption to the entire conveyor operations. Hence, there is a strong need for an effective and robust strategy and online automatic technology for the monitoring and an early diagnosis of conveyor belt mis-tracking.

An experiment and a simulation of a belt mis-tracking were performed in Ref. [15] to measure and calculate the lateral movement (i.e., mis-tracking) of conveyor belts. Computer vision was employed for monitoring conveyor belt deviation in Ref. [16] by detecting the edges of the belt and a generated laser line in the center of the belt. Ref. [17] studied the effect of belt speed, which affects belt mis-tracking, and proposed an expression to calculate the quantity of belt mis-tracking using the roller misalignment parameters. Ref. [18] proposed a real-time conveyor belt deviation detection algorithm based on a multi-scale feature fusion network. Recently, in Ref. [19], an existing self-aligning idler assembly, which was used for belt conveyors, was analyzed, and an alternate assembly was designed to overcome limitations in the existing system.

A relatively new approach to condition-based maintenance (CBM) in the form of motor current signature analysis (MCSA) has proved to be a reliable technique for monitoring motor conditions and has been the subject of considerable investigation in recent years. This is mainly because induction motors (IMs) are used in numerous applications, as they are easy to manufacture, affordable, efficient, and reliable. Moreover, compared to other diagnostic technologies, the collection of stator current data is far simpler, as no sensors need to be mounted directly on the machine, and the cost of the necessary equipment is lower. 

Among numerous technologies, higher-order spectral techniques have been shown in the literature to be effective methods for identifying faults in electromechanical systems via MCSA, Refs. [20,21,22,23]. The higher order spectra technologies, which are based on the Fourier transform, have been widely employed to diagnose nonlinearity in time-stationary processes. In Ref. [24], higher-order spectra (HOS)-based features have been used for early-stage detection of gear faults. The work reported in Ref. [25] applies conventional Fourier-based HOS in the diagnosis of distortions of aircraft engine intake airflows, referred to as intake separation. In Refs. [26,27,28], an initial application of wavelet bicoherence and wavelet cross-covariance to the diagnosis of gearbox tooth faults at an early stage and developed naturally, is presented. The HOS are used in Ref. [29] to diagnose faults for transient signals with any known nonlinear frequency variation.

Despite all the above-mentioned advantages of MCSA and all the promising results for higher-order spectral technologies, no previous work has taken advantage of these technologies for diagnosing the mis-tracking of belt conveyor systems. Ref. [30] investigated the slip and meander of belts in a belt conveyor system using motor current data. In order to examine the pattern of current fluctuation over a certain frequency range, time-frequency analysis, such as the short-time Fourier transform and the Wigner distribution, was used, i.e., the energy of a specific band of frequencies for a belt slip and meander diagnosis in a conveyor belt system. The diagnosis was achieved by comparing the energies of damaged and undamaged conditions for the specified bands. The approach and results of this paper are not applicable to belt mis-tracking diagnosis.

Therefore, based on the above literature review, there is not a fault diagnosis technology either in the existing literature or in the market that is capable of diagnosing conveyor belt mis-tracking.

This paper proposes, for the first time ever, the diagnostics of belt mis-tracking for belt conveyor systems based on MCSA. Three higher-order spectra diagnostic technologies based on the bicoherence, the tricoherence, and the cross-correlation of spectral moduli of order 3 (CCSM3) were experimentally evaluated for the first time in this study as diagnostic technologies for conveyor belt mis-tracking. The effectiveness of the considered technologies was evaluated and compared using the motor current signals, recorded under different tracking conditions, of a conveyor belt system used for baggage handling in airports at the University of Huddersfield.

The main difference between this communication and the recently published paper by the authors in Ref. [31] is that, although both pieces of research use MCSA to diagnose faults in rotating machines, neither the technologies, nor the application, the results are similar in both studies. In Ref. [31], the authors proposed two new diagnostic features in order to detect the lack of lubrication in the geared motor system: feature 1, which measured the power in a frequency bandwidth around the harmonics of the supply frequency, and feature 2, used the spectral magnitude of the fundamental harmonic of the supply frequency normalized by the average value of the spectral magnitudes of the higher harmonics of the supply frequency in the spectrum of the current signal, and characterized the non-linearity of the motor current. However, in this communication, the proposed and compared technologies were based on the bicoherence, the tricoherence, and the CCSM3, and diagnosis was performed for the mis-tracking of the conveyor belt and not for the gearmotor itself.

The main originality of this paper is a new proposition (by L. Gelman) for the first time ever in worldwide terms to use MCSA for the diagnosis of belt mis-tracking for belt conveyor systems.

Other paper novelties are as follows:

- This paper proposes for the first time a new effective technology for the diagnosis of conveyor belt mis-tracking.
- It provides novel experimental results for the diagnosis of belt mis-tracking via the proposed three technologies.
- Novel effectiveness comparisons of the proposed three technologies for the diagnosis of belt mis-tracking are carried out.

The paper contains four sections. Section 2 explains the proposed method, based on MCSA, for a belt mis-tracking diagnosis. Section 3 describes the mis-tracking experimental setup and the experiments. The effectiveness of the proposed technologies is evaluated in Section 4 using experimental data acquired from a belt conveyor system with and without a belt mis-tracking.

## 2. Diagnostic Technologies for a Belt Mis-Tracking

We propose the investigation of three diagnostic technologies to diagnose conveyor belt mis-tracking based on MCSA. When a conveyor belt is subjected to mis-tracking, friction occurs between the belt and one of the conveyor metal walls, resulting in an increase in the load of the motor that is driving the conveyor. When subjected to an increase in load, motors increase their electrical torque to compensate for the increased mechanical load, thus increasing the intensity of the supply frequency’s fundamental and higher harmonics. The suggestion of employing MCSA for such diagnostics is based on the assumption that belt friction creates changes in the harmonics of the supply frequency of the motor, which is driving the conveyor. Therefore, the proposed technologies for a belt mis-tracking diagnosis are based on the harmonics of the supply frequency. As a result of belt mis-tracking, the multiple harmonics of the supply frequency, which appear in the spectrum of motor current, are more highly correlated than in the case of normal belt tracking.

The main concept of the proposed technologies is that friction between a belt and a conveyor wall increases levels of the cross-correlations between the supply frequency’s fundamental and higher harmonics. Such an increase in the cross-correlation between the harmonics of the supply frequency could be diagnosed via higher-order spectral features, Refs. [32,33].

Thus, it is proposed that technologies of higher-order spectra could be employed for diagnosing conveyor belt mis-tracking. Three technologies of the higher order spectra: the bicoherence, the tricoherence, Ref. [34] and the CCSM3, Ref. [33], are proposed, compared, and experimentally evaluated for diagnosing a conveyor belt mis-tracking based on MCSA.

The bicoherence, or the third-order normalized cumulative spectrum, was used to estimate statistical dependencies between three phases–coupled complex spectral components in the frequency domain. The bicoherence, or the third-order normalized cumulative spectrum, is used to estimate statistical dependencies between three complex spectral components in the frequency domain. The bicoherence is a normalized version of the bispectrum, and it takes values, which are bounded between 0 and 1, and makes it convenient for quantifying damage severity. Similarly, tricoherence, the fourth-order normalized cumulative spectrum, is used to estimate statistical dependencies between four complex spectral components in the frequency domain. The tricoherence is a normalized version of the trispectrum, and it also takes values, which are bounded between 0 and 1, and makes it convenient for quantifying damage severity.

From the definitions of bicoherence and tricoherence, it is clear that the analyzed spectral components must exhibit statistical dependencies in order to obtain non-zero results, which are associated with belt conveyor faults. However, the bicoherence and the tricoherence could be used in specific cases in which faults related to spectral components follow “the bicoherence and the tricoherence frequency rules” (Refs. [34,35]). These higher-order spectra frequency requirements may not be met for all spectral components, which carry valuable information for a fault diagnosis. Therefore, these higher-order spectra frequency requirements have a limited usage of spectral components generated by faults, which could be used for a fault diagnosis via motor current signature analysis and, thus, limited fault types could be effectively diagnosed by the higher order spectra.

The cross-correlation of spectral moduli of order 3 (CCSM3) is recently proposed in Ref. [33] as a powerful tool for estimating the spectral cross-correlations between the moduli of three spectral components in the frequency domain without the need to follow “the bicoherence and the tricoherence frequency rules”. Contrary to the bicoherence and the tricoherence and other higher order spectra, the CCSM3 allows the action to take into account statistical dependencies between any needed combinations of multiple spectral components that appear due to a fault, without limitations: i.e., regardless of dependencies between their central frequencies. The first step in the estimation of the CCSM3 is to divide a current signal into overlapping or non-overlapping time segments. The second step is to perform a selected frequency transform or a selected time-frequency transform for each time segment. The third step is to estimate the moduli of the spectral components at the selected characteristic frequencies or at the selected characteristic time-frequency points. The final step is to estimate the instantaneous cross-correlations for each time segment and to average these instantaneous cross-correlations overall time segments.

The considered higher order spectral features are given, respectively by Equations (1)–(3):

(1) $$The\;bicoherence\left(f_{1},f_{2}\right)=\frac{\sum_{j=1}^{J}\left(X_{f_{1}}^{j}\right).\left(X_{f_{2}}^{j}\right).{\left(X_{f_{1}+f_{2}}^{j}\right)}^{*}}{\sqrt{\sum_{j=1}^{J}{\left(X_{f_{1}}^{j}.X_{f_{2}}^{j}\right)}^{2}}.\sqrt{\sum_{j=1}^{J}{\left(X_{f_{1}+f_{2}}^{j}\right)}^{2}}}$$

(2) $$The\;tricoherence\left(f_{1},f_{2},f_{3}\right)=\frac{\sum_{j=1}^{J}\left(X_{f_{1}}^{j}\right).\left(X_{f_{2}}^{j}\right).\left(X_{f_{3}}^{j}\right).{\left(X_{f_{1}+f_{2}+f_{3}}^{j}\right)}^{*}}{\sqrt{\sum_{j=1}^{J}{\left(X_{f_{1}}^{j}.X_{f_{2}}^{j}.X_{f_{3}}^{j}\right)}^{2}}.\sqrt{\sum_{j=1}^{J}{\left(X_{f_{1}+f_{2}+f_{3}}^{j}\right)}^{2}}}$$

(3) $$The\;CCSM3\left(f_{1},f_{2},f_{3}\right)=\frac{\sum_{j=1}^{J}\left|\left(X_{f_{1}}^{j}\right)\right|.\left|\left(X_{f_{2}}^{j}\right)\right|.\left|\left(X_{f_{3}}^{j}\right)\right|}{\sqrt[{3}]{{\sum_{j=1}^{J}\left|\left|X_{f_{1}}^{j}\right|-|\overset{-}{X_{f_{1}}}|\right|}^{3}}.\sqrt[{3}]{{\sum_{j=1}^{J}\left|\left|X_{f_{2}}^{j}\right|-|\overset{-}{X_{f_{2}}}|\right|}^{3}}.\sqrt[{3}]{{\sum_{j=1}^{J}\left|\left|X_{f_{3}}^{j}\right|-|\overset{-}{X_{f_{3}}}|\right|}^{3}}}$$

where $\overset{-}{X_{f_{k}}}=\frac{1}{J}\sum_{j=1}^{J}\left|X_{f_{k}}^{j}\right|,$ $X_{f_{k}}^{j}$ represents the short-time chirp Fourier transform, Refs. [36,37,38], at the instantaneous frequency $f_{k}$ of the current time segment $x^{j}(t)$ and J is number of segments.

The time-frequency technique, the short-time chirp-Fourier transform, was used for feature estimations instead of the Fourier transform to take into account a time variation in the supply frequency.

The experimental validation and comparison of the considered diagnostic technologies for a belt mis-tracking diagnosis are as follows.

1. Motor current signals are acquired from a belt conveyor system operating under normal tracking and mis-tracking cases, respectively (see Section 3).
2. The instantaneous supply frequency of the acquired current signals is estimated using the phase demodulation approach based on the Hilbert transform, Ref. [39]. The instantaneous supply frequency could exhibit a slight variation over time, explaining why the short-time chirp Fourier transform is used for higher-order spectral estimates instead of the Fourier transform method.
3. Instantaneous higher-order spectral components of the supply frequency are deduced from the instantaneous supply frequency and estimated in the second stage.
4. The diagnostic features [Equations (1)–(3)] are evaluated for signals corresponding to normal tracking and mis-tracking cases.

The effectiveness of the considered technologies in diagnosing mis-tracking is evaluated and compared using histograms of their diagnostic features under healthy and belt mis-tracking conditions.

**Figure 1:**
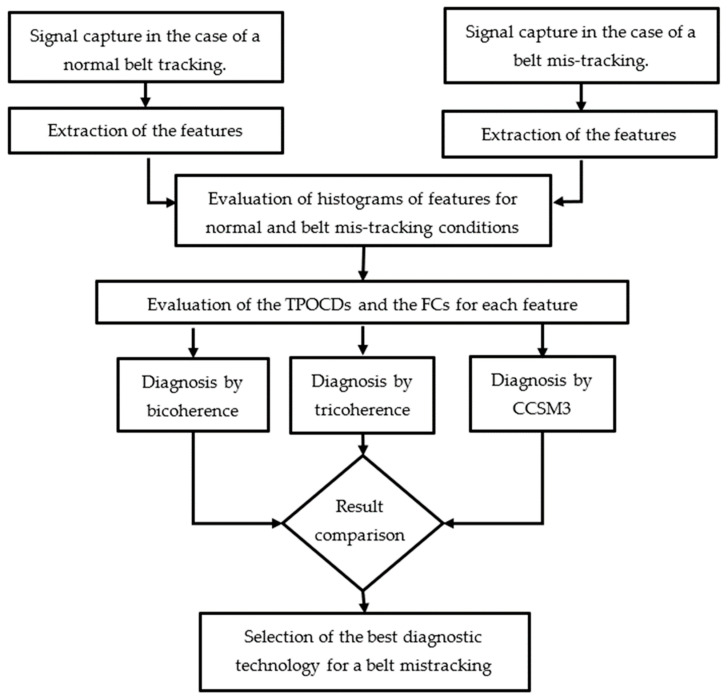
Flow chart of the adopted research method.

A flow chart of the research method, adopted to evaluate and compare the effectiveness of the considered technologies in the diagnostic of conveyor belt mis-tracking, is given in Figure 1.

The research method used in this study, as shown in Figure 1, starts by collecting signals from normal belt tracking and belt mis-tracking cases. The features of both data sets are extracted using Equations (1)–(3). The histograms of each feature for normal and belt mis-tracking conditions are created. The total probability of the correct diagnosis (TPOCD) and the Fisher criterion (FC) [40] are evaluated and compared to select the most effective diagnostic techniques for belt mis-tracking. The TPOCD is the ratio of the number of cases that are correctly diagnosed to the total number of cases. 

## 3. Experimental Setup

**Figure 2:**
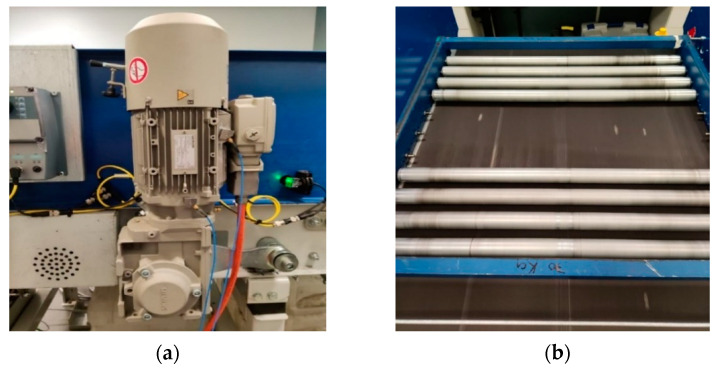
The conveyor belt system under investigation. (**a**) The gearmotor of the belt conveyor system, (**b**) The loaded belt conveyor system.

**Figure 3:**
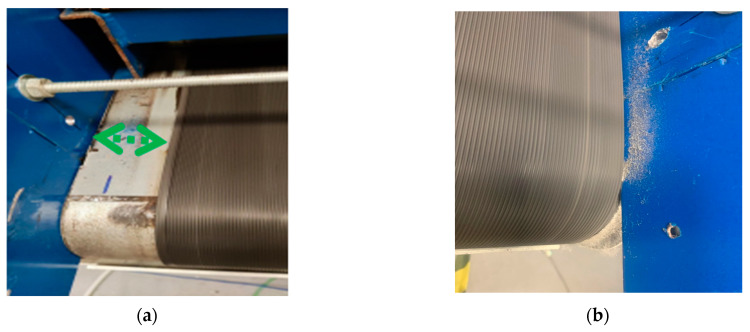
Normal belt tracking and belt mis-tracking conditions. (**a**) Normal belt tracking, drive side, no contact between the belt and the wall, (**b**) Non-drive side mis-tracking, the belt and the non-drive side wall are in contact.

A belt conveyor system used for baggage handling at airports, was considered in this study. The same type of conveyor could also be found in various other applications, making the technology proposed in this article applicable to a wide range of applications. The conveyor drive consists of a gear motor consisting of a two-stage gearbox and an AC induction motor, as shown in Figure 2a. Figure 2b shows the belt of the conveyor system loaded with a metal pallet. The motor is connected to the power line with a nominal supply frequency of 50 Hz. Henceforth, the left side of the conveyor, as shown in Figure 3a, is called the drive side because it is the side on which the gearmotor is installed. Similarly, the right side of the conveyor is called the non-drive side, as shown in Figure 3b.

The drive-side of the conveyor is shown in Figure 3a, where a suitable distance is provided between the conveyor metal wall and the belt. By contrast, Figure 3b shows the case in which the metal wall of the non-drive side is in contact with the belt (mis-tracking), resulting in friction between the belt and a metal wall.

**Figure 4:**
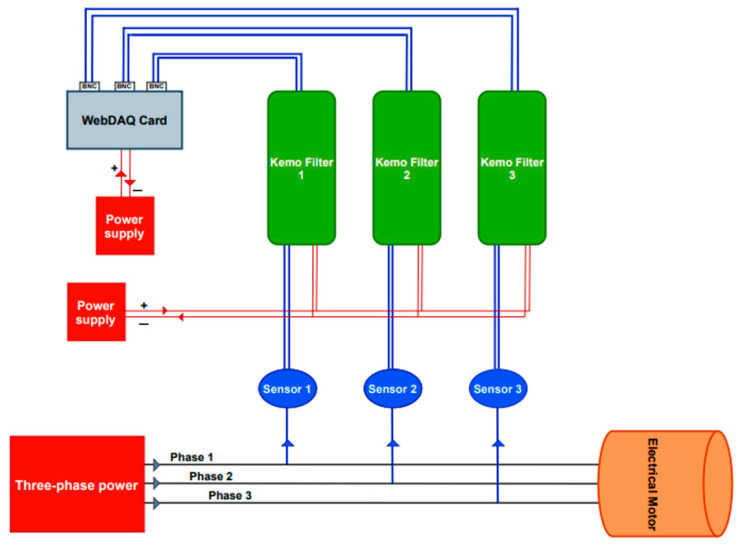
Schematic of the data acquisition system.

A schematic of the current signal acquisition system employed in this study is presented in Figure 4. LEM ATO-B10 current sensors were used to capture the motor current signals. These current sensors are from the ATO series, composed of a split-core transformer, with galvanic isolation between the primary circuit (supply circuit) and the secondary circuit (measurement circuit) utilized in measuring the stator currents.

The continuous-captured current signals were passed through KEMO amplifiers/anti-aliasing filters, which are used to amplify and limit the frequency bandwidth of the recorded signals to satisfy the Nyquist-Shannon sampling theorem. Filters with a gain of ×20 and a cut-off frequency of 8 kHz were adopted in the experiments. For the conversion of the analog signals (the KEMO filters output) into digital signals, a WebDAQ 504 data acquisition card was used, with simultaneously sampled analog inputs, 24-bit resolution, and IEPE signal conditioning.

Mis-tracking experiments were conducted under two loading conditions, namely, without load and with a load of 20 kg on the conveyor. The considered load consisted of a metallic frame with a series of rollers placed on the conveyor belt, as shown in Figure 2b.

For both loading conditions, motor current signals were recorded under normal tracking conditions and in cases of drive side and non-drive side mis-tracking, respectively, with a sampling frequency of 51,200 Hz.

At the beginning of each recording, the belt drive roller was tilted around the conveying axis by loosening or tightening its adjusting nut. This involved the fact that, during operations, the belt side to one side due to its self-centering behavior. Therefore, a mis-tracking, i.e., a contact between a belt and one of the conveyor metal walls, occurred depending on the inclination of the belt drive roller. The mis-tracking level progressively increased until it reached a maximum level; then, the belt drive roller was readjusted to a neutral (i.e., non-sloping) position, which led to a progressive decrease in the mis-tracking level until it disappeared (i.e., a return to normal belt tracking).

In total, six recordings were considered in this study, corresponding, respectively to:

- Normal belt tracking under a no-load condition.
- Normal belt tracking under a 20 kg load condition.
- Non-drive side mis-tracking under a no-load condition.
- Drive side mis-tracking under a no-load condition.
- Non-drive side mis-tracking under a 20 kg-load condition.
- Drive side mis-tracking under a 20 kg-load condition.

## 4. Results and Discussion

In this section, the diagnosis effectiveness of the considered technologies: bicoherence, tricoherence, and CCSM3, are evaluated for MCSA-based belt mis-tracking diagnosis.

Histograms of the considered diagnostic features under different tracking conditions were evaluated to assess technology effectiveness in diagnosing mis-tracking. Then, the normal probability density function (PDF) for each histogram was computed. 

The estimation of the probability density functions was conducted under the assumption of the normal distribution for features: i.e., the Gaussian probability density functions (PDF). The mathematical formula of the probability density function of a random variable X, which follows the normal distribution, could be expressed as a function of the mean μ and the standard deviation σ as follows:

(4) $$PDF\left(X\right)=\frac{1}{\sigma \sqrt{2\Pi }}e^{\frac{-{\left(x-\mu \right)}^{2}}{2\sigma^{2}}}$$

**Figure 5:**
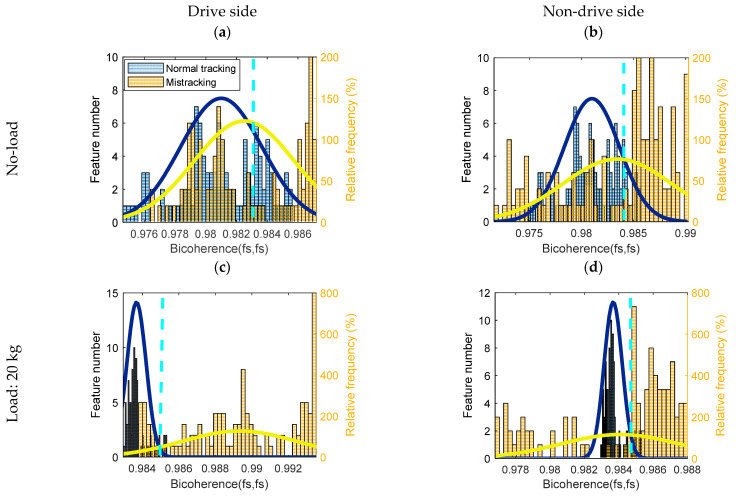
Histograms, probability density function and diagnostic thresholds represented as the aqua dotted line for the bicoherence (fs, fs) for normal and belt mis-tracking conditions: (**a**) Drive side belt mis-tracking under no-load condition, (**b**) Non-drive side belt mis-tracking under no-load condition, (**c**) Drive side belt mis-tracking under 20 kg load condition, (**d**) Non-drive side belt mis-tracking under 20 kg load condition.

**Figure 6:**
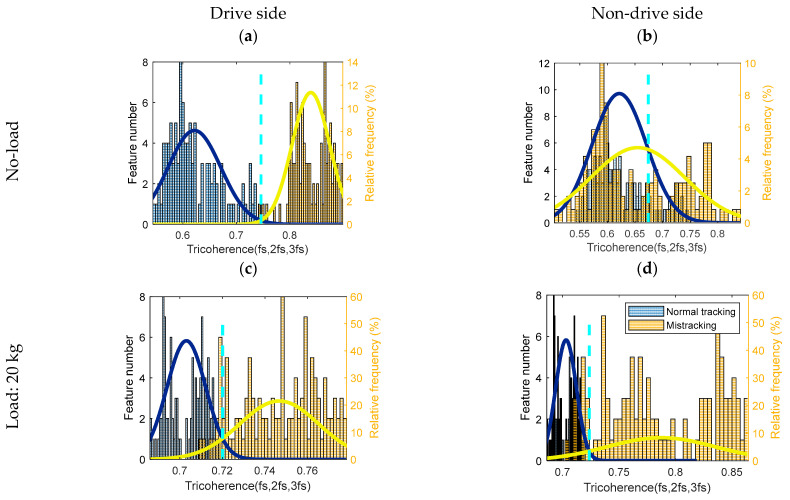
Histogram, normal power density function and diagnostic threshold represented as the aqua dotted line of the tricoherence (fs, 2fs, 3fs) for normal and mis-tracking conditions: (**a**) Drive side belt mis-tracking under no-load condition, (**b**) Non-drive side belt mis-tracking under no-load condition, (**c**) Drive side belt mis-tracking under 20 kg load condition, (**d**) Non-drive side belt mis-tracking under 20 kg load condition.

**Figure 7:**
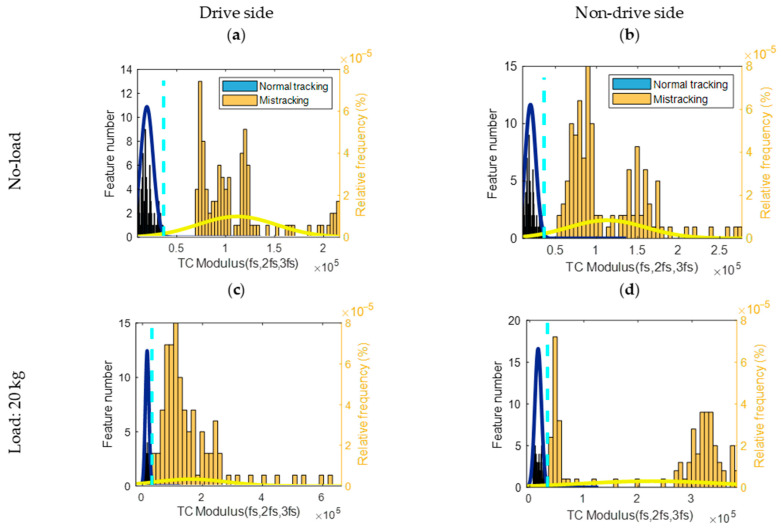
Histograms, probability density functions and diagnostic thresholds represented as the aqua dotted line of the CCSM3 (fs,2fs,3fs) for normal and mis-tracking conditions: (**a**) Drive side belt mis-tracking under no-load condition, (**b**) Non-drive side belt mis-tracking under no-load condition, (**c**) Drive side belt mis-tracking under 20 kg load condition, (**d**) Non-drive side belt mis-tracking under 20 kg load condition.

A diagnostic threshold is defined as the intersection point between two PDFs, referring to the Bayesian rule (see Figure 5, Figure 6 and Figure 7). If the feature of a normal tracking condition has a lower value than a threshold, normal tracking is correctly diagnosed. If the feature for the mis-tracking condition has a higher value than the threshold, mis-tracking is correctly diagnosed. For both loading conditions (0 and 20 kg), the CCSM3 and the magnitudes of the diagnostic features, bicoherence, and tricoherence, are estimated from the signals corresponding to normal tracking, drive side, and non-drive side mis-tracking.

Referring to Equation (2), the bicoherence (fs, fs) can be estimated for the three harmonics: the supply frequency’s fs fundamental harmonic, which is employed two times, and the supply frequency’s second harmonic. Referring to Equation (3), the tricoherence (fs, 2fs, 3fs) can be estimated for the four harmonics: the fundamental, the second, the third, and the sixth harmonics of the supply frequency fs. Referring to Equation (4), the CCSM3 can be estimated for the three harmonics: the fundamental, the second, and the third harmonics of the supply frequency fs. 

The following parameters were employed for feature estimations:

- The duration of the time segments was 20 s, i.e., the frequency resolution was 0.05 Hz.
- The overlapping between segments was 90%.
- The number of segments considered in the computing of one feature was 70.
- The time window used for the segmentation was the rectangular window.

As motor current spectra around the fundamental harmonic and the higher harmonics of the supply frequency could be enriched by multiple sidebands and harmonics related to a gearmotor, a relatively high-frequency resolution (i.e., 0.05 Hz) was selected in order to reliably isolate the supply frequency harmonics from other characteristic spectral components in the motor current spectra.

Normally, in order to obtain a reliable estimation of the higher-order spectral diagnostic features, the number of time segments for a feature averaging should be relatively high. Therefore, the selected number of segments was 70, and the overlapping between segments was 90%. In order to keep the selected frequency resolution, a rectangular time window was employed for the time segments. Histograms related to the bicoherence, tricoherence, and CCSM3 are presented, respectively, in Figure 5, Figure 6 and Figure 7.

**Figure 8:**
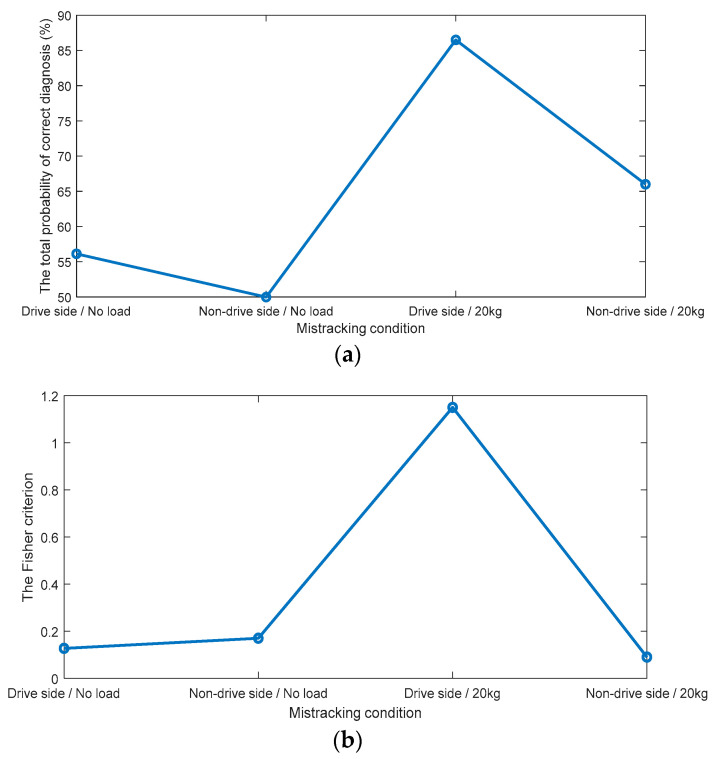
(**a**) The total probabilities of correct diagnosis (%), provided by the bicoherence (**b**) The Fisher criteria for the bicoherence.

The histograms of bicoherence values, given in Figure 5, show a clear overlap between mis-tracking conditions and the normal tracking conditions under both loading conditions (i.e., no load and 20 kg). A vertical aqua dotted line represents the diagnostic threshold, which is the intersection point between both probability density functions. The overlap was less in the case of drive-side mis-tracking under a 20 kg load condition compared to other mis-tracking cases. The total probabilities of correct diagnosis and the FCs for the bicoherence (fs, fs) are plotted in Figure 8.

According to the histograms, the best total probability of the correct diagnostics and the FC can be achieved in the case of drive side mis-tracking under a 20 kg load condition (the total probability of correct diagnosis is 86%, and the FC is 1.19), against the lower total probabilities of correct diagnosis, and the lower FCs for other cases. Even though the diagnosis accuracy reached 86% in the case of a drive side mis-tracking under a 20 kg load, the overall total probability of correct diagnostics (average for all cases) obtained by the bicoherence (fs, fs) was 67%, making it a non-accurate diagnosis technology for a belt mis-tracking.

**Figure 9:**
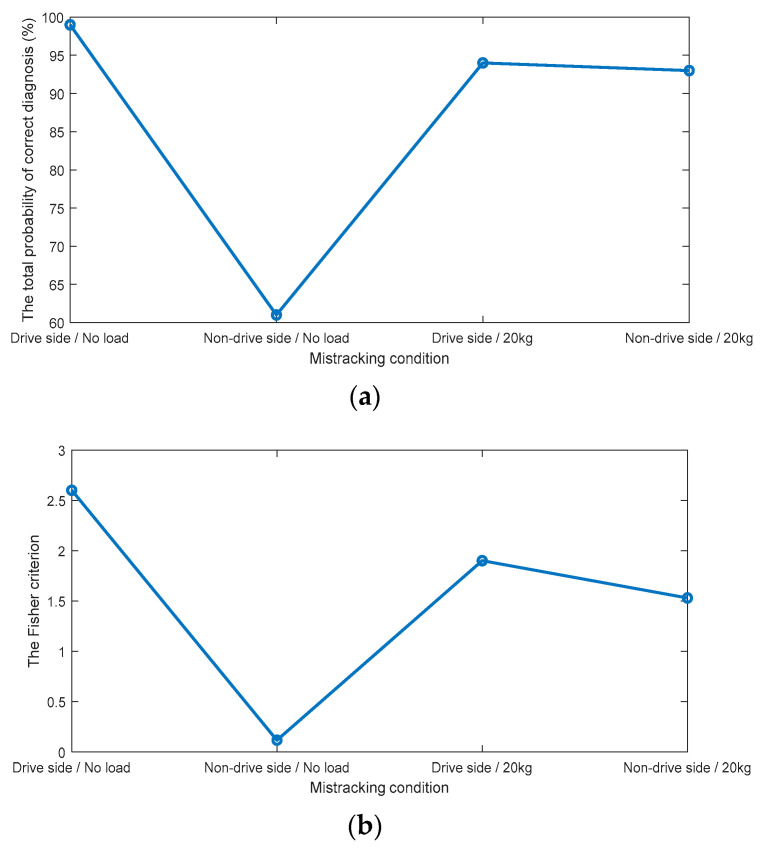
(**a**) The total probabilities of correct diagnosis (%), provided by the tricoherence (**b**) The Fisher criteria for the tricoherence.

The tricoherence histograms, shown in Figure 6, reveal that: (i) under no-load condition, while separation was noted between the normal tracking and drive side mis-tracking features, a complete overlap was obtained between the normal tracking and non-drive side mis-tracking data; (ii) under a 20 kg load condition, a slight overlap was noted between the normal tracking data and the two mis-tracking cases, i.e., the drive side mis-tracking and the non-drive side mis-tracking. The total probabilities of correct diagnosis and the FCs of the tricoherence (fs, 2fs, 3fs) are plotted in Figure 9.

The total probability of the correct diagnostics of the tricoherence was higher than 93% for three out of the four mis-tracking cases, i.e., the drive side mis-tracking under no-load condition and both drive side and non-drive side mis-tracking under a 20 kg load condition. In the case of non-drive side mis-tracking under a no-load condition, the total probability of correct diagnostics by tricoherence was 61%. The average (for the four cases) total probability of correct diagnostics, provided by the tricoherence technology, was 86%. Therefore, the tricoherence technology could also be considered a non-accurate diagnosis technology for belt mis-tracking.

**Figure 10:**
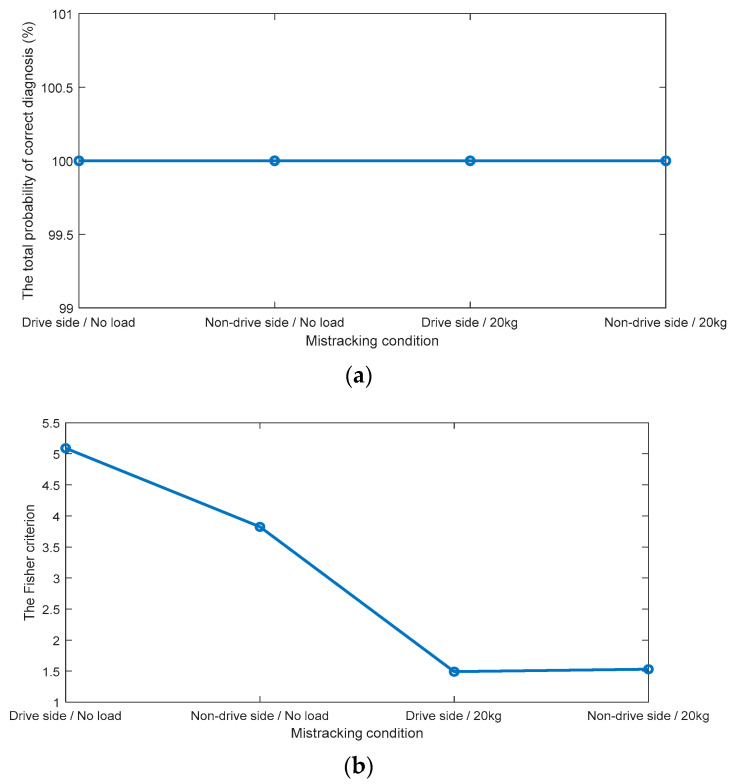
(**a**) The total probabilities of correct diagnosis (%), provided by the CCSM3 (**b**) The Fisher criteria for the CCSM3.

The CCSM3 diagnostic results shown in Figure 7 show a complete separation between the distributions of the four mis-tracking cases with respect to normal tracking. This is translated in Figure 10 by 100% estimates of the total probability of correct diagnostics for all studied cases. The probability density functions of the CCSM3 features for the mis-tracking conditions have higher variances than for the normal tracking features, which can be explained by the fact that multiple levels of a belt mis-tracking were considered in the mis-tracking experiments. The CCSM3 technology is suggested as a highly effective diagnosis technology for belt mis-tracking under all the considered conditions.

The novel comparison between the CCSM3 technology, the tricoherence technology, and the bicoherence technology shows that the proposed CCSM3 technology allows the following essential increases in the average total probability of correct diagnostics:

1. A total of 33% compared with the bicoherence technology.
2. A total of 14% compared with the tricoherence technology.

These novel comparison results justify that it is more beneficial to have diagnostics via higher-order spectral technologies and exclude the phase spectra from spectral harmonics, which are involved in the estimation of higher-order spectral technologies.

**Figure 11:**
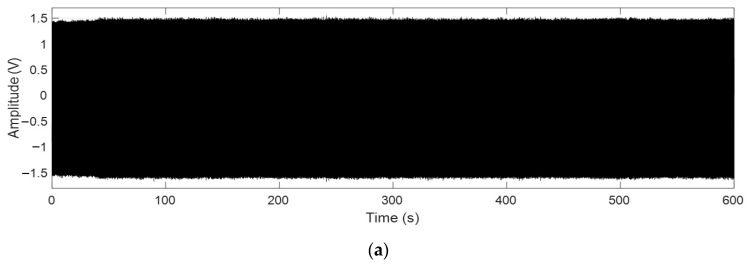

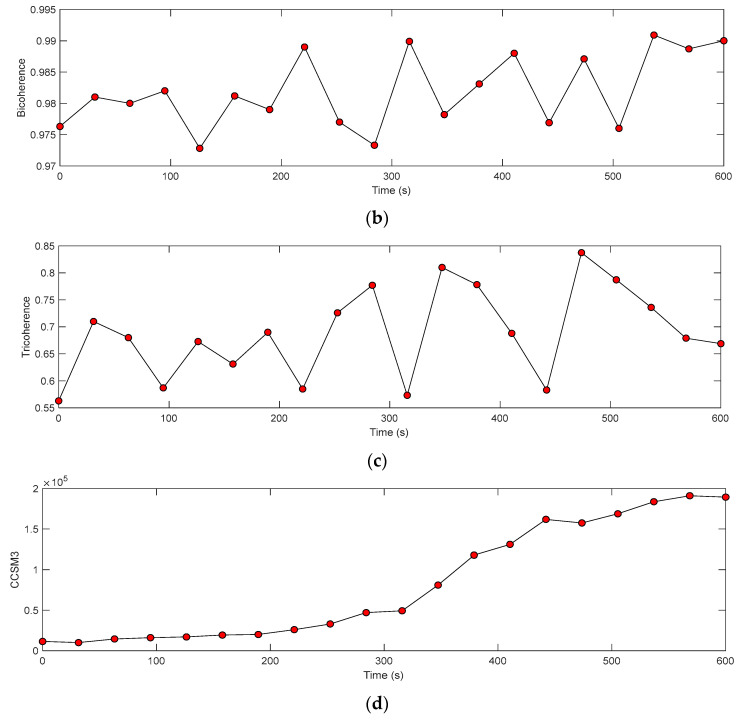
(**a**) A time signal of a motor current recorded during a transition from a normal tracking to a non-drive side mis-tracking, (**b**) The estimated bicoherence values, (**c**) The estimated tricoherence values, (**d**) The estimated CCM3 values.

A time domain signal of a motor current was recorded during a transition from normal belt tracking to a non-drive side belt mis-tracking of the conveyor belt under a no-load condition, as presented in Figure 11a. Figure 11b–d shows the respective values of the bicoherence, the tricoherence, and the CCSM3 estimated for the time domain signal in Figure 11a.

In the considered case, a belt mis-tracking event started at time 200 s. Figure 11 clearly shows that among the considered three technologies (i.e., the bicoherence, the tricoherence, and the CCM3), only the CCM3 technology showed a clear smooth transition of feature values between the normal belt tracking and belt mis-tracking, which additionally confirmed its effectiveness in diagnosing conveyor belt mis-tracking. On the other hand, even though the overall trend of the bicoherence and the tricoherence increased in the case of belt mis-tracking compared to the normal belt tracking condition, a clear overlap between the calculated values of the bicoherence and the tricoherence for both conditions could be observed in Figure 11, confirming their low efficiency in diagnosing belt mis-tracking.

Based on the overall obtained results, the CCSM3 technology is suggested as a powerful effective technology for conveyor belt tracking fault diagnosis. Compared to the existing technologies, the CCSM3 technology has the advantage that it can be applied by acquiring the motor current signals without the need to install sensors outside the conveyor’s electrical box. Indeed, conveyors are most often used in harsh environments, which makes the installation of sensors on a conveyor chain very difficult or even impossible. In addition, unlike other diagnostic technologies, the collection of motor stator current data can offer the possibility of diagnosing various possible conveyor faults, not only belt mis-tracking. This is possible using various MCSA-based technologies, as reviewed in Ref. [1]. The minor disadvantage of the proposed technology is a relatively high computational complexity related to a diagnostic feature estimation, which could be easily overcome by powerful IT tools.

## 5. Conclusions

1. Based on the literature review, there is no fault diagnosis technology either in the existing literature or in the market capable of diagnosing conveyor belt mis-tracking.
2. It was proposed for the first time in worldwide terms that motor current signature analysis for the diagnosis of conveyor belt mis-tracking could be employed.
3. The current signals of an AC induction gearmotor, driving a belt conveyor system for baggage handling in airports, were recorded while the conveyor was operating under two loading conditions (no-load and 20 kg load), respectively, for the cases of normal tracking, drive side mis-tracking, and non-drive side mis-tracking.
4. Three higher-order spectral technologies based on the bicoherence, the tricoherence, and the cross-correlation of spectral moduli of order 3 (CCSM3) were comprehensively experimentally evaluated for the diagnostics of conveyor belt mis-tracking. The tested technologies employed the supply frequency fundamental and the higher harmonics as characteristic spectral components.
5. The bicoherence, the tricoherence and the CCSM3 of the supply frequency fundamental, and the higher harmonics were extracted from the current signals corresponding to the normal tracking and two distinct types of mis-tracking conditions: i.e., the drive side mis-tracking and the non-drive side mis-tracking.
6. The effectiveness of the proposed technologies was estimated via the total probabilities of the correct diagnosis and the Fisher criteria. It was obtained that:
  - The bicoherence diagnosis technology is a non-accurate technology for belt mis-tracking.
  - The tricoherence diagnosis technology is also a non-accurate diagnosis technology for belt mis-tracking.
  - The CCSM3 technology is suggested to be a highly effective diagnosis technology for belt mis-tracking as it provides 100% estimates of the total probability of correct diagnostics under all considered mis-tracking conditions.

A novel comparison between the CCSM3 technology, tricoherence technology, and bicoherence technology shows that the proposed CCSM3 technology allows the following essential increases in the average total probability of correct diagnostics:

- A total of 33% compared with the bicoherence technology.
- A total of 14% compared with the tricoherence technology.

Based on the obtained results, CCSM3 is suggested to be a powerful technology for conveyor belt tracking fault diagnosis with the advantage that it can be applied simply by acquiring the motor current signals. The minor disadvantage of the proposed technology is a relatively high computational complexity related to a diagnostic feature estimation, which could easily be overcome by powerful IT tools.

7. This study is foremost for a conveyor belt mis-tracking diagnosis. The proposed novel conceptualization and the novel online automatic diagnosis technology could make a considerable impact on a conveyor belt mis-tracking diagnosis for baggage handling systems at airports, belt conveyors for the mining industry, and belt conveyor-based handling systems to move goods, products, raw goods, and other materials for various manufacturing industries, transportation industry, food industry, etc.

## Data Availability

Not applicable.
